# Ursolic Acid—A Pentacyclic Triterpenoid with a Wide Spectrum of Pharmacological Activities

**DOI:** 10.3390/molecules201119721

**Published:** 2015-11-19

**Authors:** Łukasz Woźniak, Sylwia Skąpska, Krystian Marszałek

**Affiliations:** Department of Fruit and Vegetable Product Technology, Institute of Agricultural and Food Biotechnology, 36 Rakowiecka Street, 02-532 Warsaw, Poland; skapska@ibprs.pl (S.S.); marszalek@ibprs.pl (K.M.)

**Keywords:** ursolic acid, anticancer, triterpene, anti-inflammatory, antibacterial

## Abstract

Ursolic acid (UA) is a natural terpene compound exhibiting many pharmaceutical properties. In this review the current state of knowledge about the health-promoting properties of this widespread, biologically active compound, as well as information about its occurrence and biosynthesis are presented. Particular attention has been paid to the application of ursolic acid as an anti-cancer agent; it is worth noticing that clinical tests suggesting the possibility of practical use of UA have already been conducted. Amongst other pharmacological properties of UA one can mention protective effect on lungs, kidneys, liver and brain, anti-inflammatory properties, anabolic effects on skeletal muscles and the ability to suppress bone density loss leading to osteoporosis. Ursolic acid also exhibits anti-microbial features against numerous strains of bacteria, HIV and HCV viruses and *Plasmodium* protozoa causing malaria.

## 1. Introduction

**Figure 1:**
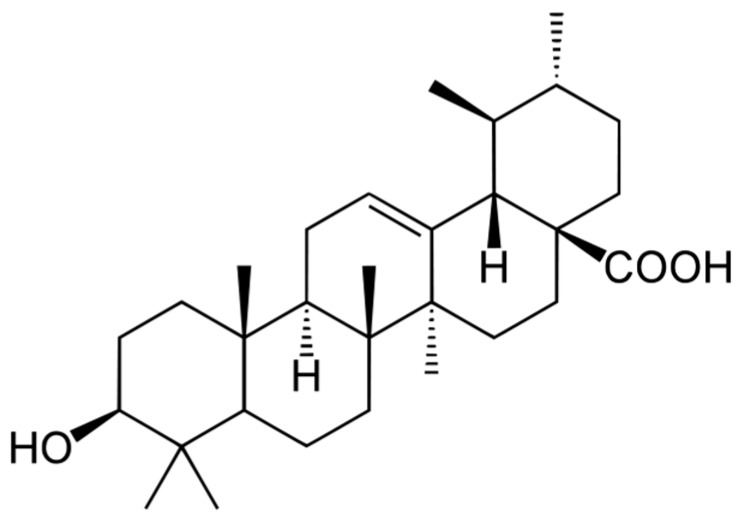
Structure of ursolic acid.

Ursolic acid (3β-hydroxy-urs-12-ene-28-oic acid, UA, Figure 1) is a pentacyclic terpenoid exhibiting a wide range of pharmaceutical properties. Ursolic acid is a secondary plant metabolite, usually present in the stem bark, leaves or fruit peel. The health promoting activities of this compound have been unknowingly used for centuries—as an ingredient of herb extracts employed in folk medicine. In recent years researchers, looking for natural biologically active substances, came back to this source of knowledge acquired over generations.

This review is an attempt to describe the present state of knowledge about the pharmacological properties of ursolic acid. Some of the biological activities of this compound have been reviewed before, but these papers were focused on singular aspects [1,2,3,4] or rapidly became dated due to the immense interest in the features of UA in recent years [5,6,7].

## 2. Natural Occurrence and Biosynthesis of Ursolic Acid

Ursolic acid and related triterpene compounds like oleanolic acid, betulinic acid, uvaol or α- and β-amyrin are widespread in plants. Their content and composition differs between various species, due to the presence and activity of the enzymes responsible for their synthesis. Amongst plants matrices with a high content of ursolic acid and of potentially practical significance as a source of this compound one can mention apple (*Malus domestica*) fruit peel, marjoram (*Origanum majorana*) leaves, oregano (*Origanum vulgare*) leaves, rosemary (*Rosmarinus officinalis*) leaves, sage (*Salvia officinalis*) leaves, thyme (*Thymus vulgaris*) leaves, lavender (*Lavandula angustifolia*) leaves and flowers, eucalyptus (*Eucalyptus*) leaves and bark, black elder (*Sambucus nigra*) leaves and bark, hawthorn (*Crataegus* spp.) leaves and flowers, coffee (*Coffea arabica*) leaves and the wax layer of many edible fruits [8,9].

**Figure 2:**
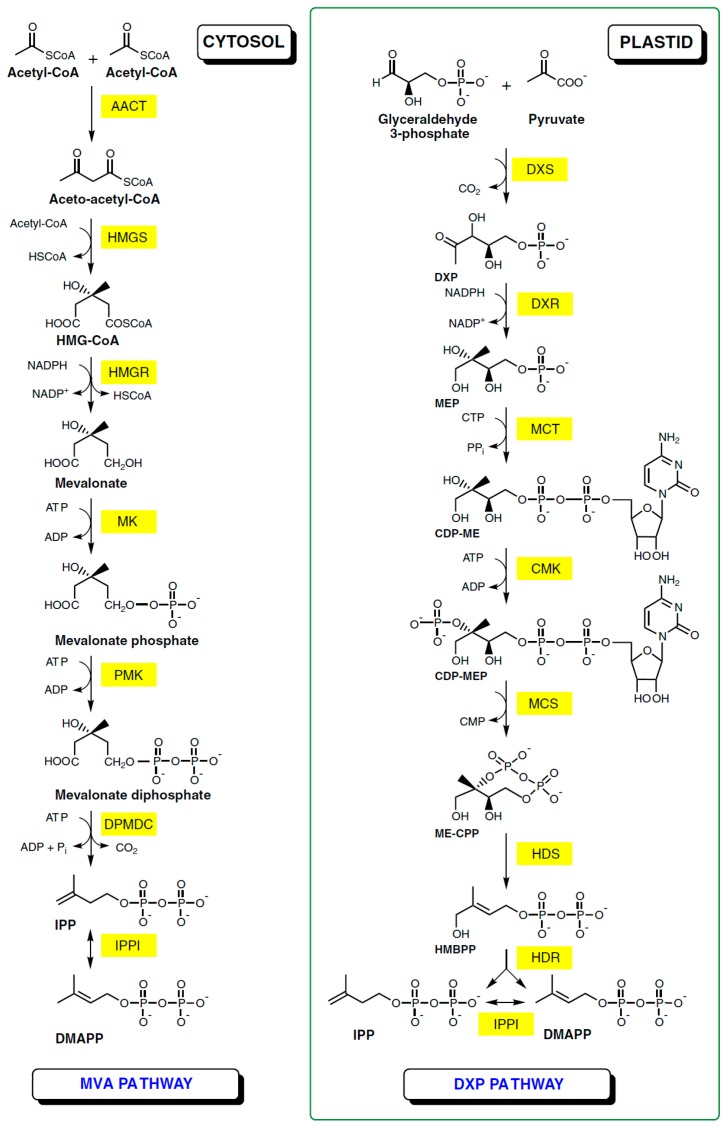
IPP formation by the MPA and DXP pathways [12].

The biosynthesis of ursolic acid and related compounds in plant tissues should be considered as a three phase process. The first stage is the production of isopentenyl diphosphate (IPP) which is a five-carbon building block utilized to create all terpenic compounds. For many years it has been believed that the mevalonate pathway (MVA) is the exclusive source of this compound. In this cytosol-carried metabolic pathway two molecules of Acetyl-CoA (created in the citric acid cycle) are transformed to one molecule of IPP through a six stage process. Recent investigations have discovered another route, the deoxyxylulose/methylerythritiol phosphate (DXP) pathway. In this plastid-located process isopentyl diphosphate is synthesized from pyruvate and glyceraldehyde-3-phosphate (Figure 2). Synthesis of triterpenes in the plastid is impossible due to the lack of necessary enzymes, however the possibility of cross-talk between the two presented pathways is considered [10,11].

The second stage of UA production is synthesis of 2,3-oxidosqualene and its cyclisation leading to formation of α-amyrin. Molecules of IPP and its isomer dimethylallyl diphosphate (DMAPP) are used to create squalene (through the intermediates geranyl pyrophosphate and farnesyl pyrophosphate). Then squalene epoxidase oxidizes this compound to 2,3-oxidosqalene. The group of enzymes named oxidosqualene cyclases (OSCs) is responsible for the cyclisation and rearrangement of the terpenoid chain leading to the formation of various scaffolds, including α-amyrin [13].

The last stage is modification of α-amyrin by group of cytochrome P450 enzymes called α/β-amyrin 28-monooxygenases. The methyl group-containing C-28 is oxidized to a carboxyl thus finishing the UA biosynthesis process [13].

## 3. Ursolic Acid as a Tool in Cancer Prevention and Therapy

### 3.1. General Review of Literature Data on Ursolic Acid Anti-Cancer Activities

Ursolic acid is one of the most promising substances of biological origin when it comes to the prevention and therapy of cancer. Novel pharmacological strategies do not rely only on the destruction of tumor cells, but also modulate their metabolism to prevent angiogenesis and metastasis, enforce differentiation of cells and protect healthy tissues against inflammation and oxidative stress that may lead to neoplasm formation.

UA can be described as a multi-tasking agent; it influences several cell signaling enzymes and simultaneously protects it against carcinogenic agents. The summary of the studies describing ursolic acid’s impact on carcinomas *in vitro* and *in vivo* is shown in Table 1. While papers reporting protective effects (including anti-inflammatory and antioxidant properties) are listed in Table 2.

It should be noted that in the majority of mentioned works the authors were testing pure compounds or attributed therapeutic properties to ursolic acid. There are also numerous studies describing the effects of plant extract without assigning the activity to a particular compound; the authors did not include them in this work.

### 3.2. Cellular Signaling Pathways and Enzyme Inhibition—The Key to the Ursolic Acid Activity against Cancer

**Figure 3:**
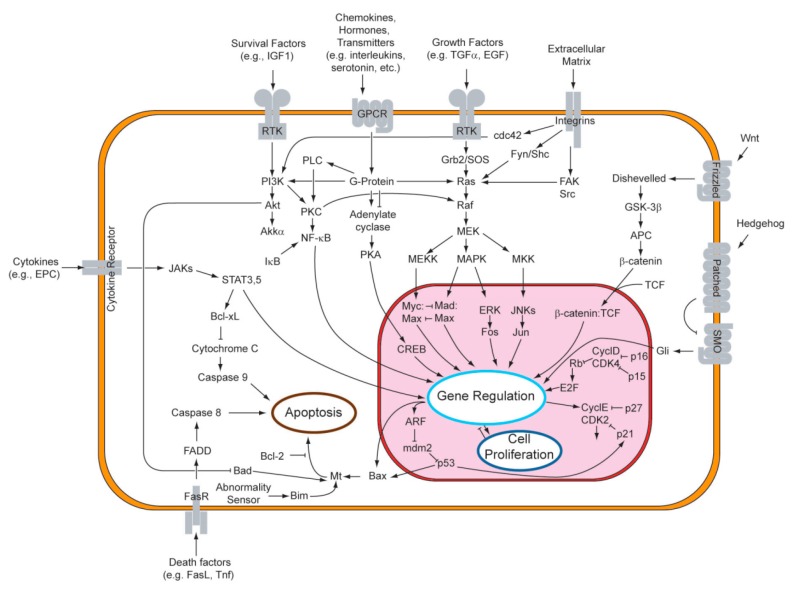
Main intracellular signaling pathways. From: *Wikipedia*. Wikimedia Foundation, Inc.

To fully understand modus operandi of anti-cancer drugs one must take a closer look at cell signaling. This very complex system of communication coordinates all cellular activities and responses on extracellular signals. The schematic diagram of the main intracellular signal routes is presented in Figure 3.

**Table 1:** Ursolic acid in cancer therapy.

Carcinoma Type	Model Used	Mechanism of Action
bladder cancer	cell lines (NTUB1 and T24)	induction of apoptosis: (i) using endoplasmic reticulum stress response to activate c-Jun N-terminal kinase signaling [14] (ii) connected with reactive oxygen species production [15]
breast cancer	rodent model (mice)	inhibition of tumor growth and induction of apoptosis by modulation of PI3K/Akt/mTOR pathway signaling [16]
	cell lines (MCF-7, MCF-7/ADR and MDA-MB-231)	inhibition of growth [17] antiproliferative activity [18] suppression of migration and metastasis by modulating c-Jun N-terminal kinase (JNK), Akt and mTOR signaling [19] induction of apoptosis: (i) via mitochondrial death pathway and extrinsic death receptor pathway [20] (ii) by suppressing expression of FoxM1 protein [21] cytotoxicity [22]
cervical cancer	cell lines (HeLa and SiHa)	inhibition of proliferation [23,24] induction of apoptosis through mitochondrial intrinsic pathway and suppression of ERK1/2 MAPK pathway [25] enhancement of chemotherapeutic efficiency [26] cytotoxicity [22,27]
colorectal cancer	cell lines (Caco-2, CO115, CT26, DLD1, HCT15, HCT116, HT29, SW480 and SW620)	inhibition of proliferation [17,18,28,29,30] induction of apoptosis: (i) via downregulation of Bcl-2, Bcl-xL and survivin activity [17] (ii) by influencing PI3K signaling pathway [28] (iii) via p53-independent upregulation of death receptors [31,32] (iv) by autophagy through JNK pathway [33] through cyclooxygenase 2 (COX-2) pathway [34] enhancement of ionizing radiation-induced apoptotic effect [35] cytotoxicity [36]
fibrosarcoma	cell lines (HT1080)	suppression of metastasis by downregulation of matrix metallopeptidase 9 (MMP-9) [37]
gastric cancer	cell lines (AGS, BGC823, SGC7901 and SNU-484)	induction of apoptosis: (i) via downregulation of Bcl-2 [38] (ii) by activation of caspase-3, -8, and -9 and downregulation of Bcl-2 expression [39] (iii) through inhibition of cyclooxygenase 2 [40] cytotoxicity [22,36] suppression of proliferation [24]
glioma	rodent model (rats)	inhibition of metastasis through suppressing association of ZIP/p62 with PKC-ζ and downregulation of MMP-9 [41]
	cell lines (1321N1, U87 and U251)	inhibition of proliferation and induction of apoptosis by suppression of TGF-β1/miR-21/PDCD4 pathway [42] promotion of differentiation by inhibition of the endogenous reverse transcriptase (RT) [43] suppression of growth via reactive oxygen species accumulation [44]
hepatic cancer	rodent model (mice)	suppression of AMF/PGI mediated tumorigenic activities [45] inhibition of proliferation and induction of apoptosis by downregulation of cyclooxygenase-2 (COX-2) [46]
	cell lines (H22, Hep3B, HepG2 and Huh7)	antiproliferative activity [18,24,47] induction of apoptosis: (i) by activation of caspase-3, -8, and -9 and downregulation of Bcl-2 expression [19] (ii) through downregulation of XIAP and mitochondrial-dependent pathway [48] (iii) via downregulation of survivin and activation of caspase-3 through PI3K/Akt/mTOR pathway [49] antiangiogenic properties [50] cytotoxicity [36]
melanoma	rodent model (mice)	antiangiogenic properties by changing matrix metalloproteinases activity [51]
	cell lines (A375, B16F10 and M4Beu)	induction of apoptosis: (i) through mitochondrial intrinsic pathway and caspase-3 activation [52] (ii) by activation of p53 and caspase-3 gene expression and suppression of NF-κB mediated activation of Bcl-2 [53] (ii) through mitochondrial pathway [54] inhibition of proliferation and promotion of differentiation by suppression of the endogenous reverse transcriptase (RT) [43] enhancement of ionizing radiation-induced apoptotic effect [31]
leukemia	cell lines (Jurkat, HL60, HL60/ADR, K562, K562/ADR, THP1 and U937)	induction of apoptosis: (i) through downregulation of ezrin [55] (ii) via upregulation of PTEN gene expression and inactivation of PI3K/Akt/mTOR pathway [56] (iii) by inactivation of PKB as well as activation of JNK [57] involving enhanced intracellular Ca2+ signals [58] inhibition of growth [17] inhibition of proliferation [23] induction of differentiation by ERK1/2 MAPK pathway activation [59] cytotoxicity [22]
lung cancer	cell lines (A549, ASTC-a-1, Calu-6, H640 and H3255)	inhibition of proliferation [23,60] inhibition of metastasis by suppressing expression of AEG-1 and inhibition of NF-κB [61] enhancement of chemotherapeutic effect [26] induction of apoptosis by upregulation of matrix metalloproteinase and activation of caspase-3 [62]
lymphoma	cell lines (Daudi)	induction of apoptosis [63]
multiple myeloma	cell lines (U266, RPMI and 8226.MM1.S)	suppression of proliferation and chemosensitization, inhibition of STAT3 activation pathway by expression of tyrosine phosphatase SHP-1 protein [64]
neuroblastoma	cell lines (IMR32 and SH-SY5Y)	inhibition of proliferation [23,24]
ovarian cancer	cell lines (CAOV and SK-OV-3)	inhibition of proliferation by suppressing ERK activity and expression of ERK 1/2 [65] induction of apoptosis: (i) by upregulation of BAX (Bcl-2-like protein 4) expression and downregulation of Bcl-2 expression [65] (ii) by activation of caspases and phosphorylation of GSK3 beta [66]
pancreatic cancer	cell lines (AsPC-1, Capan-1, MIA, Paca-1 and PANC-2)	induction of apoptosis: (i) with upregulation of p53, p21(waf1) and Noxa proteins levels [67] (ii) by induction of JNK pathway and suppression of PI3K/Akt/NF-κB pathway [68]
prostate cancer	rodent model (mice)	suppression of metastasis by inhibition of CXCR4/CXCL12 signaling [69] suppression of growth by downregulation expression of cyclin D1 and COX-2 and upregulation of caspase-3 levels [70]
	cell lines (DU145, LNCaP and PC3)	induction of apoptosis: (i) through activation of caspases and downregulation of c-IAPs [71] (ii) through autophagy [72] (iii) via activation of JNK and inhibition of Akt pathways [73] (iv) through cyclooxygenase 2 (COX-2) pathway [34] (v) by activation of JNK-induced Bcl-2 phosphorylation and degradation [74] (vi) by downregulation of Bcl-2 [75] enhancement of ionizing radiation-induced apoptotic effect [31] cytotoxicity [36]
thyroid cancer	cell lines (ARO)	inhibition of proliferation and promotion of differentiation by inhibition of the endogenous reverse transcriptase (RT) [43]

**Table 2:** Ursolic acid in cancer prevention.

Preventive Effect	Model Used	Mechanism of Action
anti-inflammatory	mouse primary splenocytes	inhibition of Th2 cytokines production [76]
	activated T cells, B cells and macrophages; mice	suppression of NF-κB, AP-1 and NF-AT activity [77]
	rat edema tests	unclear, probably connected with glucocorticoids [78]
	mice	reductions of Th2 cytokines and ovalbumin-specific IgE production, and eosinophil infiltration via the Th2-GATA-3, STAT6, and IL-17-NF-κB pathways [79]
	human intestinal epithelial cells and peritoneal macrophages from mice	inhibition of production of pro-inflammatory cytokines, IκBα phosphorylation/degradation and NF-κB DNA binding activity [80]
	rat mast cells	inhibition of histamine release [81]
	murine peritoneal macrophages	suppression of NO production and iNOS expression via downregulation of NF-κB activation; attenuation of expression of COX-2 and the secretion of proinflammatory cytokines like TNF-α and IL-6 [82]
	PC12 cells	attenuation of H_2_O_2_ and MPP-induced release of IL-6 and TNF-α [83]
	oedema in mice	attenuation of inflammation [84]
	pleurisy in mice	reduction of leukocytes, interleukin-1 beta (IL-1β) and tumor necrosis factor-alpha (TNF-α) levels [85]
	phagocyte cells	inhibition of histamine release; inhibition of prostaglandins and leukotrienes production [86]
	arthritis-induced mice	alteration of Th1/Th2 cytokine production [87]
	Th17 cells	suppression of interleukin-17 production by antagonizing function of RORγt protein [88]
	biochemical assays	inhibition of cyclooxygenase-2 catalyzed prostaglandin biosynthesis [89]
anti-oxidative	PC12 cells	attenuation of H_2_O_2_ and MPP-induced impairment in catalase and superoxide dismutase activity [83]
	rat liver microsomes	protection against lipid peroxidation [90]
	RAW247 cells	inhibition of NO production [91]
	isolated rat heart mitochondria	decrease in H_2_O_2_ production in the mitochondria [92]
	human blood lymphocytes	normalization of antioxidant levels; reduction of lipid peroxidation [93]
	Caco-2 cells	protection of DNA against oxidative damage [94]
chemical-induced cancer	mouse skin	inhibition of binding benzo(a)pyrene and 7,12-dimethylbenz(a)anthracene to DNA [95]
	rats	suppression of preneoplastic lesions formation by 1,2-dimethylhydrazine [96]
	rats	inhibition of formation of aberrant crypt foci by azoxymethane [97]
	human bronchial epithelial cells and mice	inhibition of tobacco smoke extract-induced cell injury [98]
radiation-induced cancer	mice	enhancement of hematopoietic system recovery [99]
ROS-induced cancer	murine T cells	inhibition of cell activation through modulation of NF-κB signaling [100]
	rats	attenuation of hepatocellular carcinoma induction by diethylnitrosamine-induced reactive oxygen species [101]
	keranocite cell line and mice	skin cancer prevention; protection against hydrogen peroxide induced DNA damage [102]
viral-induced cancer	Raji cell line and mice	inhibition of Epstein-Barr virus activation induced by 12-O-tetradecanoylphorbol-13-acetate (TPA) [103,104,105]

The anticancer activity of ursolic acid is associated with its ability to influence the activity of several enzymes. Therefore it is able to modulate processes occurring inside tumor cells activating routes leading to cell death (usually by apoptosis) and suppressing ones leading to the proliferation, growth and migration of cancer.

#### 3.2.1. Signaling Pathways

The MAPK/ERK and PI3K/AKT/mTOR signaling cascades play critical roles in the transmission of signals from growth factor receptors to regulate gene expression. Both these pathways are responsible for anti-apoptotic and drug resistance effects in cells [106]. The ability to suppress communication through these routes is one of the most important anti-cancer activities of UA. It has been reported by numerous research teams in various tumor types.

High expectations are also surrounding UA impact on nuclear factor κB. Activity of NF-κB is connected with reaction on such stimuli as cytokines, free radicals or antigens, and it plays crucial role in immunologic answer against infection. Malignant cells are characterized by abnormally high activity of this transcription factor, what leads to intense proliferation and make NF-κB one of the main targets of modern oncotherapy [107]. Capability to lower activity of NF-κB has been noticed not only by oncologist but also scientists dealing with bone and brain issues.

#### 3.2.2. FOXM1 Transcription Factor

Forkhead box (FOX) proteins are family of transcription factors playing crucial role in regulating expression of genes involved in cell growth. FOXM1 has been recognized as exceptional important as its aberrant upregulation might be inducing genomic instability and leading to malignant transformation [108]. FOXM1 has been already used as biomarker of early stages of cancer. This protein has been also shown to possess ability to cross-talk with other molecules playing role in cancer development (like: NF-κB, COX-2, ERK and MMPs) [109]. Inhibition of Forkhead box M1 expression by UA has been reported by Wang *et al.* [21] in their study on MCF-7 human breast cancer cells.

#### 3.2.3. Apoptosis Regulating Proteins

Apoptosis is the process of programmed cell death occurring as a result of activation of the specific cellular pathways. In contrast to necrosis, this process is highly regulated and leads to chromosomal DNA fragmentation. The induction of apoptosis by various agents is an important part of modern cancer therapies. Unfortunately apoptosis in cancer cells is often blocked by the activity of mutated genes regulating the cell cycle. Therefore different steps of the apoptotic process should be targeted to bypass such blocks [1].

Apoptosis induction is the uppermost anti-cancer activity of ursolic acid. It has been reported in dozens of papers, as regards several cancer types, both *in vitro* and *in vivo*. This aptitude is often connected with the Bcl-2 apoptosis regulators activity. This group of evolutionarily related proteins consist of both pro- and anti-apoptotic agents and is regarded as crucial in regulation of cell death through intrinsic apoptotic pathway [110]. Ursolic acid is able to change activity of three amongst 25 members of this family: anti-apoptotic proteins Bcl-2 and Bcl-xL are suppressed, while pro-apoptotic BAX activity is enhanced [17,19,38,39,65].

Caspases are family of cysteine proteases playing essential role in apoptosis. They are final step of cell death pathways and are responsible for e.g., DNA fragmentation, cleavage of nuclear proteins and as a result blebbing and cell death [111]. Inhibitors of apoptosis proteins (IAPs) are a family of functionally and structurally related proteins which are able to bind caspases and consequently prevent apoptosis of cell. Overexpression of these compounds can be responsible for drug resistance of neoplasm cells and its inhibition became one of the therapeutic targets in cancer treatment [112]. Ursolic acid exhibits ability to reduce expression of proteins from IAP family. Amongst influenced molecules researchers mentioned XIAP [48], c-IAPs [71] and survivin [17,49].

#### 3.2.4. Endogenous Reverse Transcriptase

Sequencing of the human genome shown that retrotransposable elements make up about 45% of the human DNA. Almost all of these elements contain genes responsible for reverse transcriptase (RT) coding. In most tissues expression of RT-coding genes is very low, however high expression is distinctive for undifferentiated cells like embryos, germ cells or tumor cells. Sciamanna *et al.* [113] revealed that RT inhibitors had been able to reduce cell proliferation and induce morphological differentiation in four carcinoma lines. They also connected RT activity with control of proliferation and differentiation in neoplastic cells.

Role of ursolic endogenous reverse transcriptase as a mediator of ursolic acid properties was reported by Bonaccorsi *et al.* [43]. Their work confirmed ability of UA to suppress growth and induce differentiation in cancer cells; it also shown that ursolic acid exhibited RT inhibiting activity and connected it with anti-tumor activities.

#### 3.2.5. Factors Involved in Metastasis and Angiogenesis

Angiogenesis is the formation of new blood vessels from other pre-existent ones during development, growth, wound repair or the female reproductive cycle. Angiogenesis is one of cancer’s hallmarks since it is required for both tumor progression and dispersal of metastatic cells. This resulted in the fact that the inhibition of angiogenesis has become an alternative therapeutic approach to cancer therapy. The angiogenic process is activated by intracellular signals that activate resting endothelial cells, which are stimulated to release degrading enzymes allowing endothelial cells to migrate, proliferate, and finally differentiate to form new vessels. Any of these steps might be a potential target for pharmacological compounds [114].

Anti-angiogenic properties of ursolic acid are usually attributed to inhibition of the downregulation of matrix metalloproteinases activity. Metalloproteinases are group of the enzymes involved in degradation of extracellular matrix. Their activity in tumor tissues is elevated due to increased demand for oxygen and glucose of neoplasm. UA inhibiting activity against MMP-9 has been confirmed by several research teams, however activity against MMP-2 remains subject of discussion: Huang *et al.* [62] reported that UA is able to suppress MMP-2 expression while Cha *et al.* [37] did not observe such an effect in their study.

#### 3.2.6. Cyclooxygenase-2 (COX-2)

The connection between inflammation and cancer had been suggested as early as in 1863 by Rudolf Virchow. Currently chronic inflammation, with concomitant activity of cytokines and increased production of reactive oxygen species, is recognized as a cancerogenesis-promoting condition [115]. The cyclooxygenases are enzymes responsible for conversion of arachidonic acid to prostaglandin H2, which is precursor of other prostanoids. Their inhibitors (e.g., acetylsalicylic acid, ibuprofen, naproxen) are often used as anti-inflammatory drugs and painkillers [116]. Ursolic acid proved to be efficient COX-2 inhibitor able to suppress inflammation progress [80,86]. Additionally lowering activity of this cyclooxygenase has been correlated with caspase-3 activity and affected apoptosis rate in cancer cells [40,46].

### 3.3. Protection against Tumor-Inducing Agents

Several tests of the anti-carcinogenic activity of ursolic acid against different induction sources have been conducted. These test included chemical agents (such as benzo(a)pyrene, azoxymethane and tobacco smoke extract) [95,96,97,98], reactive oxygen species producers (such as hydrogen peroxide) [100,101,102], ionizing radiation [99] and viral pathogens (Epstein-Barr virus) [103,104,105].

### 3.4. Ursolic Acid as a Drug—Clinical Trials

The ultimate goal of every cancer research is the implementation of the compound to clinical use. Currently ursolic acid is undergoing phase I trials to evaluate its safety and adverse effects in patients. Due to poor water solubility and low bioavailability ursolic acid had been administered as a liposomes. So far results of only three such studies have been published [117,118,119]—all of them were performed in The People’s Republic of China. Ursolic acid liposomes showed tolerable toxicity and adverse effects—only one of 108 patients reported third grade adverse activity. The most frequent complaints were nausea, diarrhea and skin problems. The common conclusion of all studies was the necessity of the continuation of research during phase II tests.

## 4. Impact of Ursolic Acid on Condition and Functioning of Body Organs

### 4.1. The Liver

The liver is one of the most important organs of the body. It is responsible for a wide range of metabolic functions, including detoxification of xenobiotics, production of hormones and digestive enzymes, glycoside and fat-soluble vitamins storage and the decomposition of red blood cells. Due to its strategic location and multidimensional functions the liver is prone to many diseases, like hepatitis, hepatic steatosis, cirrhosis, cholelithiasis and drug-induced liver damage. Fortunately liver is the only internal organ capable to regenerate—as little as 25% of the original mass can reconstruct its full size.

Ursolic acid showed good protective activity against a wide range of liver-threatening substances. Saraswat *et al.* [120] were testing UA isolated from *Eucalyptus tereticornis* extract against ethanol toxicity in isolated rat hepatocytes. They found that this triterpene was able to decrease the loss of hepatocyte viability by as much as 76%. A similar problem has been studied by Saravanan *et al.* [121], this time however *in vivo* using alcohol-administered rats. They reported that UA increased the level of circulatory antioxidants and serum protein and decreased the total bilirubin level and lipid peroxidation markers. Histopathological observations were in correlation with biochemical parameters. Paracetamol and tetrachloride were other liver-intoxicating agents tested by Shukla *et al.* [122] and Martin-Aragón *et al.* [123], respectively. Both these papers revealed increase of hepatocytes viability and improvement of serum markers in UA treated samples.

The impact of UA on metabolic disorders in high fat diet-fed mice and rats was surveyed in research conducted by Sundaresan *et al.* [124] and Li *et al.* [125]. The first group focused on hepatic lipid accumulation and noticed that the combined treatment of UA and rosiglitazone (an antidiabetic drug in the thiazolidinedione class) significantly reduced the hepatic marker enzyme activities and decreased the lipid accumulation in the liver. Furthermore, combination treatment downregulated lipogenic genes and upregulated fatty acid oxidative genes. The latter team discovered that UA effectively ameliorated high fat diet-induced hepatic steatosis through a PPAR-α involved pathway, via improving key enzymes controlling lipid metabolism.

Wang *et al.* [126] were looking for a possibility to improve hepatic fibrosis patients by UA administration. They performed tests on hepatic stellate cells (HSCs) isolated from rats and found that UA induces apoptosis of these cells, leading to partial amelioration of fibrosis.

Interesting results were acquired by Jin *et al.* [127]. They discovered that ursolic acid was able to enhance liver regeneration after partial hepactomy in mice. A significant increase in liver to body weight ratio has been observed (compared to control group), along with the stimulation of expression of cyclins and C/EBP proteins.

### 4.2. The Heart

Cardiovascular diseases are the major causes of mortality and morbidity in industrialized countries. They are responsible for about 30% of all deaths worldwide. Amongst the most common disorders of the cardiovascular system one can mention myocardial infraction (commonly known as heart attack), stroke, atherosclerosis, hypertension and varicose veins. Although not all cardiovascular diseases are life threatening, all of them significantly decrease life quality and generate enormous social and financial costs [128].

The first study which reported the impact of UA on the cardiovascular system was conducted by Somova *et al.* [129]. It revealed that the administration of UA has been able to lower the heart rate of genetically hypertensive rats by 32%.

Further research has been carried out in various directions. Vasorelaxant properties of ursolic acid were investigated by Aguirre-Crespo *et al.* [130], Rios *et al.* [131] and Shimada and Inagaki [132]. The first two teams connected the activity of UA with the production and release of nitric oxide (NO) in isolated thoracic aorta and *in vivo* on Wistar rats, respectively. Shimada and Inagaki focused on the inhibitory effect on angiotensin I-converting enzyme (ACE), which plays an important role in the regulation of blood pressure.

Ursolic acid has been also used as a compound with a potent protective effect in artificially induced (by isoproterenol administration) myocardial infarction. Senthil *et al.* [133] were testing the level of cardiac markers, lipid peroxidation products, lipid profiles and membrane-bound proteins in the serum of Wistar rats treated with isoproterenol. They found that UA was able to prevent alterations and restore enzyme activity to normal levels indicating cardioprotective activities. These results were confirmed and expanded in two works by Radhiga *et al.* [134,135]. It was reported that UA had been able to stabilize the level of numerous markers and blood constituents. In addition the anti-apoptotic effect on cardiac muscle cells has been shown. Heart-protective features of UA were also presented in work by Saravanan and Pugalendi [136]. They investigated oxidative stress induced in ethanol-administered rats. Like in the abovementioned papers ursolic acid lowered levels of lipid peroxidation products and increased the activities of free radical scavenging enzymes and antioxidant levels in heart tissue.

Administration with ursolic acid also prevents injuries to blood vessels. Pozo *et al.* [137] revealed that UA in daily doses of 6 mg/kg body weight was able to inhibit neointima formation in rats’ carotid artery. In the paper by Lv *et al.* [138] the authors describe the protective effect of UA on the human umbilical vein endothelial cells (HUVECs) damaged by C-reactive protein. They reported that UA inhibited the harmful effect in a dose-dependent manner.

The impact of ursolic acid on atherosclerosis is the subject of dispute amongst scientists since some studies show potentially beneficial effects while others show potentially negative effects [139]. For example Ullevig *et al.* [140] reported the inhibition of monocyte dysfunction and the slowing down of accelerated atherosclerosis in diabetic mice, while Messner *et al.* [141] described the stimulation of atherosclerotic plaque formation in mice administered with UA.

The potentially harmful effect of UA intake has been presented by Kim *et al.* [142]. They discovered that this triterpene is capable to make platelets more susceptible to aggregation, and they should be used with caution by people with a predisposition to cardiovascular events.

### 4.3. The Brain

Excitotoxicity and oxidative stress are two phenomena that have been repeatedly described as being implicated in a wide range of disorders of the nervous system. Such ailments include several common idiopathic neurological diseases, traumatic brain injury, and the consequences of exposure to certain neurotoxic agents. Both excitoxicity and oxidative stress result from the failure of normal compensatory mechanisms to maintain cellular homeostasis and may lead to permanent damaging of the brain and decrease of cognitive functions [143].

The first research focusing on the protective effect of ursolic acid on neurons has been conducted by Shih *et al.* [144]. Neuronal cultures from rats’ hippocampi were treated with kainic acid with and without pretreatment of 5–15 µM ursolic acid. It was observed that UA significantly decreased damage and suppressed free radical generation.

Lu *et al.* [145] were investigating the protective effect of UA against galactose-induced damage on mice brains. They reported an increase of antioxidant enzymes activity (catalase, superoxide dismutases, glutathione peroxidase and glutathione reductase) and linked it with triterpene activity. Furthermore, they found that UA significantly increased the level of the growth-associated protein GAP43. The next paper of this team [146] reported that ursolic acid administration significantly improved the behavioral performance of D-gal-treated mice in the step-through test and the Morris water maze task. The results also showed that UA significantly reduced the number of activated microglia cells and astrocytes, downregulated the expression of NOS and COX-2, and decreased the interleukin and tumor necrosis factor. Moreover, UA significantly inhibited NF-κB nuclear translocation in the prefrontal cortex of galactosis-treated mice.

Suppressing NF-κB by ursolic acid as a method to attenuate cognitive deficits and avoid brain damage has been reported by Wang *et al.* [147] and Li *et al.* [148]. Their models were lipo-polysaccharide-damaged mouse brains and mice after cerebral ischemia, respectively.

Wu *et al.* [149] described another intracellular signaling pathway involved in UA neuroprotective activity. They discovered that UA can activate PI3K/Akt signaling and suppress Forkhead box protein O1 (FoxO1) activity in domoic acid-induced mice. Moreover, UA attenuated the mitochondrial dysfunction and cognitive deficits through promoting Akt phosphorylation and FoxO1 nuclear exclusion in the hippocampus.

The impact of UA on the brain is not limited to the cellular level. Machado *et al.* [150], encouraged by results acquired during a test utilizing rosemary extract, surveyed the influence of UA on mice behavior. They performed two predictive tests of antidepressant properties: the tail suspension test (TST) and the forced swimming test (FST). The compound increased immobility time in both tests. Further investigation showed that activity of UA was likely mediated by interaction with the dopaminergic system.

Research conducted by Colla *et al.* [151] confirmed these results using TST and open-field test. Interactions with other neuromodulators showed an involvement of the serotonergic and noradrenergic systems, but not the glutamatergic or opioid systems, in the antidepressant-like effect of UA. Later work by the same team [152] revealed that ursolic acid exhibited some anxiolytic-like activities. Mice administered with UA performed open field test, elevated plus maze test, light/dark box test and marble burying test. The results show that ursolic acid (10 mg/kg) elicited an anxiolytic effect in the first and second test.

### 4.4. Skeletal Muscles

Skeletal muscles contraction powers the human body’s movements and is essential for maintaining stability. Muscle tissue accounts for almost half of the human body mass and, in addition to its power-generating role, is a crucial factor in maintaining homeostasis. Given its central role in human mobility and metabolic function, any deterioration in the contractile, material, and metabolic properties of skeletal muscle has an extremely important effect on human health.

The term sarcopenia originates from the Greek words sarx (flesh) and penia (loss) and is used to describe the degenerative loss of muscle mass (atrophy) and its quality associated with aging. This expression is used to describe both: cellular processes (denervation, mitochondrial dysfunction, inflammatory and hormonal changes) and their outcomes such as decreased muscle strength, decreased mobility and function, increased fatigue and reduced energy needs. In addition, reduction of muscle mass in aged individuals has been associated with decreased survival rates following critical illnesses. It is estimated that sarcopenia affects more than 50% of people aged 80 and older [153].

To develop potential therapy against skeletal muscle atrophy Kunkel *et al.* [154] identified mRNA sequences regulated by fasting in human and mice muscles. These expression signatures were analyzed with Connectivity Map database and ursolic acid has been selected from over 1300 bioactive molecules as the one with the biggest anti-atrophic potential. Subsequent *in vivo* test on mice confirmed these capacities. Orally administered UA induced muscle hypertrophy, reduced denervation-induced muscle atrophy and changed the gene expression in muscles. Researchers connected triterpene activity with enhancing insulin/IGF-1 (insulin-like growth factor) signaling. A later paper by the same team [155] reports that UA increased Akt activity, as well as downstreamed mRNAs that promote glucose utilization (hexokinase-II), blood vessel recruitment (Vegfa) and autocrine/paracrine IGF-I signaling. As a result, skeletal muscle mass, fast and slow muscle fiber size, grip strength and exercise capacity were increased.

Bakhtiari *et al.* [156] were investigating if ursolic acid was able to rejuvenate skeletal muscles *in vitro* and *in vivo*. They found that UA elevated the expression of anti-aging genes SIRT1 (*ca.* 35 folds) and PGC-1a (*ca.* 175 folds). *In vivo* tests on a mice model revealed a decreased level of cellular energy charges (such as ATP and ADP) and increased proliferation and neomyogenesis in muscle cells. The authors draw the conclusion that UA can be considered as a potential candidate for the treatment of pathological conditions associated with muscular atrophy and dysfunction, such as skeletal muscle atrophy, amyotrophic lateral sclerosis (ALS) and sarcopenia.

The direct impact of UA on muscle strength was surveyed by a Korean team led by Bang [157]. Sixteen healthy male participants were divided into two groups (UA/placebo) and were performing resistance training for 8 weeks. Their characteristics, blood parameters and muscle strength were measured pre- and post-experiment. A significant increase in all muscle strength parameters was observed in the UA group, as well as a decrease of body fat and growth of IGF-1 and irisin in blood. The parameters of the placebo group remained unchanged.

### 4.5. Bones

Bone is a dynamic tissue that undergoes continual adaptation during life to attain and preserve skeletal size, shape and structural integrity. It consists of highly specialized cells, mineralized and unmineralized connective tissue matrix, and spaces that include the bone marrow cavity, vascular canals, canaliculi, and lacunae. When the skeleton reaches maturity, its development continues in the form of a periodic replacement of old bone with new at the same location. This process is called remodeling and is responsible for the complete regeneration of the adult skeleton every 10 years. The purpose of remodeling in the adult skeleton is not entirely clear, although in bones that are load bearing, this process most likely serves to repair fatigue damage and to prevent excessive aging and its consequences. Several types of cells are involved in the remodeling process, but the two most important are osteoblasts (bone-forming cells) and osteoclasts (cells able to remove mineralized bone matrix) [158].

The activity of osteoblasts and osteoclasts is crucial for maintaining proper bone structure and is regulated by differentiation from mesenchymal precursor cells and apoptosis. Several factors (e.g., sex steroid deficiency, senescence and glucocorticoid excess) can cause an imbalance between excessive osteoclastogenesis and inadequate osteoblastogenesis. The result is bone loss leading to osteopenia and osteoporosis [159]. Pharmacological treatment includes two categories: anti-resorptive agents (e.g., calcitonin or hormone replacement therapy) and anabolic agents (e.g., parathyroid analogues) [160].

Lee *et al.* [160] were the first to study the impact of ursolic acid on bone formation. They found that UA induces the expression of osteoblast-specific genes with the activation of mitogen-activated protein kinases, nuclear factor NF-κB and activator protein-1. Another important outcome of their research was proving ursolic acids’ bone-forming activity *in vivo*, in a mouse calvarial bone.

Tan *et al.* [161] found that triterpenic fraction from loquat (*Eriobotrya japonica*) was able to significantly decrease bone mineral density in oviarectomized mice by inhibiting osteoclast production. Dose-depended inhibitory effect of the extract on the differentiation of osteoclasts without any cytotoxicity was observed. A later paper by this research team [162] presents the results of an investigation of the structure-activity relationship of loquat extracts. It shows that, amongst 18 triterpenoids, ursolic and pomolic acid showed particularly strong inhibitory activity.

The possible mechanism of osteoclastogenesis inhibition by UA was evaluated by Jiang *et al.* [163]. The results indicated that UA can effectively suppress mRNA and protein expression by inhibiting NF-κB signaling and partially c-Jun N-terminal kinase signaling. Likewise, UA induced dose dependent attenuation of titanium particle-induced mouse calvarial bone loss. In conclusion, these results demonstrate that UA protects against wear particle-induced osteolysis by suppressing osteoclast formation and function.

Yu *et al.* [164] were investigating the impact of UA on the bone deleterious effect in streptozotocin-induced diabetic mice. Their results confirmed earlier observations: the enhancement of osteogenesis and the suppression on osteoclast differentiation. Research conducted by Fu *et al.* [165] was also carried out on diabetic-induced mice, however in this instance the aim was inhibiting tryptophan hydroxylase 1 (TPH1), one of the enzymes responsible for bone loss. Researchers synthesized several UA derivatives and investigated their inhibitive properties, obtaining molecules with improved features.

### 4.6. Other Organs

So far the influence of ursolic acid on other organs has been described only in a limited number of papers. The impact on skin has been investigated by two teams. One led by Both [166] has been testing liposome-encapsulated UA activity towards restoring and maintaining ceramide and collagen content in skin. Results showed that UA has been able to increase both the ceramide content of cultured normal human epidermal keratinocytes and the collagen content of cultured normal human dermal fibroblasts. In addition, UA liposomes increased the ceramide content of the skin of human subjects, with the increase occurring after only 3 days of treatment. On the other hand results of a study by Wójciak-Kosior *et al.* [167] present UA as a potent endangerment to skin. Ursolic and oleanolic acid activity against human skin fibroblasts (HSF) has been compared, showing that UA had exhibited much higher cytotoxic activity towards HSFs and thus it should not be used in dermal products.

Ding *et al.* [168] and Pai *et al.* [169] conducted research on the nephroprotective activity of UA. The teams were using aristolochic acid intoxicated zebrafish and gentamycin administrated rats, respectively. Both studies confirmed UA’s ability to decrease chemically-induced damage to kidneys.

The protective effect of ursolic acid has been also tested by Chen *et al.* [170] in lipopolysaccharide-induced acute lung injury in a mouse model. UA markedly reduced lethality, improved survival time and decreased lung pathological changes. The results suggested that UA is capable of improving survival times in LPS-induced acute lung injury.

## 5. Anti-Microbial Properties of Ursolic Acid

### 5.1. Anti-Bacterial Activity

The fight against bacterial infections is one of the most important tasks of medicine. The development of antibiotics in the 1940s gave physicians a powerful tool against infections and has saved the lives of millions of people. However, because of the widespread and sometimes inappropriate use of these substances, strains of antibiotic-resistant bacteria have begun to emerge. These newer, stronger bacteria pose a significant threat to human health and a challenge to drug researchers. Therefore, there is a continuous search for new, safe antimicrobial agents, including those from natural sources. A number of researches has been performed to evaluate the anti-bacterial properties of ursolic acid and related compounds (Table 3). Some of them concerned *in vitro* determination of minimal inhibition concentration (MIC) of UA and other triterpenes against different strains of bacteria [171,172,173,174]. Others, like the work of Kurek *et al.* [175] focused on the specific effects of UA on bacterial metabolism (in this case the impact on peptidoglycan metabolism).

**Table 3:** Anti-microbial and anti-parasitic activity of ursolic acid.

Species	References
**Bacteria**	
*Aeromonas caveae*	[171]
*Bacillus cereus*	[171,172]
*Bacillus sphaericus*	[173]
*Bacillus subtilis*	[173,176]
*Enterococcus faecalis*	[177]
*Escherichia coli*	[171,176,177]
*Klebisiella pneumoniae*	[171]
*Listeria monocytogenes*	[171,175,178]
*Mycobacterium tuberculosis*	[179,180,181]
*Pseudomonas aeruginosa*	[171,177]
*Pseudomonas syrinagae*	[173]
*Ralstonia solanacearum*	[175]
*Shigella flexneri*	[171]
*Staphylococcus aureus*	[171,176,177,182,183,184]
*Staphylococcus epidermis*	[175]
*Streptococcus mutans*	[185,186,187]
*Streptococcus pneumoniae*	[178,183]
*Streptococcus sobrinus*	[186]
*Streptomyces scabies*	[176]
*Vibrio cholerae*	[171]
**Viruses**	
Human immunodefiency virus	[188,189,190,191,192]
Hepatitis C virus	[189,193,194]
Herpes simplex virus	[195]
**Protozoa**	
*Leishmania amazonensis*	[196]
*Plasmodium falciparum*	[197,198,199,200]
*Trypanosoma brucei rhodesiense*	[197]
*Trypanosoma cruzi*	[201]
**Fungi**	
11 species	[202]
**Nematodes (Roundworms)**	
*Brugia malayi*	[203]
*Wuchereria bancrofti*	[203]

Ursolic acid activity against tuberculosis-causing *Mycobacterium tuberculosis* has been investigated by Woldemichael *et al.* [179] and Jiménez *et al.* [180]. They proved the activity of triterpenes (including UA) extracted from plants of known pharmaceutical activity—*Calceolaria pinnifolia* and *Chamaedora tepejilote*. Further research by Jiménez-Arellanes *et al.* [181] confirmed that UA can be used against drug-resistant strains of these bacteria.

The ability to overcome bacterial resistance against antibiotics was also tested by Horiuchi *et al.* [182] who focused their work on vancomycin-resistant *Enterococci* and Kim *et al.* [183] who used methicillin-resistant *Staphylococcus aureus*. Both studies showed that UA can be used simultaneously with antibiotics to enhance their activity.

Hu *et al.* [174] conducted research on sepsis induced by cecal ligation and puncture. They found that injections of UA were able to significantly improve survival and attenuate lung injury of infected rats.

The impact of orally delivered ursolic acid on intestinal microbiota was studied by Feng *et al.* [204]. Their results showed that it decreased the microbial diversity in the proximal intestine while having an opposite effect in the distal intestine. UA also inhibited colonization by energy harvest-related microbes and might be able to enhance intestinal health by inhibiting colonization by *Proteobacteria*.

### 5.2. Anti-Viral Properties

The human immunodeficiency virus (HIV) and human hepatitis C virus (HCV) infections are chronic and wide-spread illnesses that represent serious public health problems. According to a 2012 UNAIDS report on the global AIDS epidemic, about 34 million people were living with HIV, 2.5 million had acquired new HIV infections and 1.7 million had died of HIV-related causes worldwide during 2011.

HIV-1 protease is a retroviral aspartyl protease that is essential for the life-cycle of HIV. It cleaves newly synthesized polyproteins at the appropriate places to create the mature protein components of an infectious HIV virion. Due to its importance in metabolism this enzyme became the prime target for drug therapy. In 1996 Quere *et al.* [192] found that ursolic acid can act as an inhibitor of this protease, with IC_50_ near 1 µM. The expected mechanism of action was dimerization inhibition. Kashiwada *et al.* [190] confirmed these results and compared the activity of various triterpenes. Subsequent works focused mainly on inhibiting HIV-1 protease using plant extracts rather than pure compounds, due to its lower price and easier access [188,189,191].

It is also estimated that about 3% of the global population is infected with the hepatitis C virus. Chronic hepatitis C infection is the leading cause of cirrhosis, hepatocellular carcinoma and liver transplantations in developed countries [189]. Anti-HCV properties of UA were discovered recently. Kong *et al.* [193] reported that triterpenes are responsible for the anti-viral activity of the Chinese herb *Ligustrum lucidum*. Virus spreading was inhibited, at least partly, by suppressing NS5B RNA-dependent RNA polymerase. Garcia-Risco *et al.* [194] investigated extracts of heather (*Calluna vulgaris*) and they confirmed UA activity against human hepatitis virus C.

Research conducted by Bag *et al.* [195] showed that UA is an active agent responsible for the anti-herpes activity of *Mallotus peltatus* extract. Investigators claimed that UA was probably inhibiting the early stage of multiplication and can be used as an anti-HSV agent.

### 5.3. Activity against other Microbes and Parasites

Malaria is the parasitic disease with the greatest impact—it affects around 40% of the world’s population, spanning across more than 100 countries. Its etiological agent is a protozoa belonging to the genus *Plasmodium*.

In 2006 two independent research teams led by van Baren [197] and Cimanga [200] discovered that triterpenes (including UA) were responsible for the anti-malarial activity of extracts from *Satureja parvifolia* and *Morinda lucida*. Innocente *et al.* [198] and Della-Veccia *et al.* [199] developed UA derivatives and evaluated them by their anti-Plasmodium activity. Despite using different modification methods both teams acquired compounds with very high anti-malarial properties.

Yamamoto *et al.* [196] investigated the impact of UA on mice infected with *Leishmania amazonensis*. The curative effect of triterpene-rich fraction was similar to amphotericin B (often used in clinical therapy), however the dose required to eliminate microbes was smaller. Moreover, triterpenic fraction did not cause microscopic alterations in the liver, spleen, heart, lung, and kidney of the experimental groups.

The influence of UA on the treatment of *Trypanosoma cruzi* infections was reported by de Silva Pereirra *et al.* [201] They found that oral administration of UA can significantly reduce the parasitic peak during the acute phase of infection.

Ursolic acid, betulinic acid and six of their derivatives were tested against eleven mucocutaneous and cutaneous mycotic agents [202]. The MIC values of the piperazinyl derivatives against pathogenic yeast were in the range of 16–32 µg/mL.

The activity of UA was also examined against parasites. Saini *et al.* [203] evaluated the activity of UA extracted from *Nycanthes arbor-tristis* against *Brugia malayi* and *Wuchereria bancrofti*—tropical filariae responsible for elephantiasis. They discovered that UA was able to induce apoptosis of these nematodes by downregulating and altering the level of some key antioxidants.

## 6. Conclusions

Ursolic acid is a widespread compound of plant origin exhibiting wide range of the pharmacological activities. The biggest attention amongst scientists has been captured by the role that UA can play in treatment and prevention of cancer. Amongst other intriguing features of this triterpene anti-microbial properties and protective effect on internal organs against chemical-damage should be mentioned. However some studies point out negative effects of administration of this compound, suggesting that impact of UA on human’s health in some cases can be compared to the double-edged sword. Analysis of literature indicates that various effects can be linked to one phenomenon. The example might be inhibition of NF-κB activity, which leads to cancer cells apoptosis, anti-inflammatory effects and bone-forming activity.
